# Acquired Brown Syndrome After a Fist Punch: A Case Report

**DOI:** 10.7759/cureus.31975

**Published:** 2022-11-28

**Authors:** Saif S Alobaisi, Azam I Alromaih, Khaled A Alabduljabbar, Abdulmalik A Alyahya, Nawaf M Alanazi

**Affiliations:** 1 Pediatric Ophthalmology Department, King Abdullah Specialist Children Hospital, Riyadh, SAU; 2 College of Medicine, King Saud Bin Abdulaziz University for Health Sciences, Riyadh, SAU; 3 Ophthalmology Department, King Khaled Eye Specialist Hospital, Riyadh, SAU; 4 Ophthalmology Department, ​Al-Iman General Hospital, Riyadh, SAU

**Keywords:** surgery, ocular motility disorders, pediatric, trauma, acquired brown syndrome

## Abstract

A 15-year-old male presented with double vision in the left and upward gaze following a hit in the right orbital region. The orthoptic assessment revealed -2 limitation of elevation in the adduction position of the right eye and right hypotropia of 20 prism diopter (PD) in the left gaze and right hypotropia of 10 PD in the upward gaze. He was diagnosed with traumatic Brown syndrome and planned for superior oblique lengthening surgery for the right eye. Two months postoperatively, the patient has a normal extra-ocular motor function with the elimination of diplopia and significant improvement of elevation of the right eye in the adduction position. Herein, we discuss the clinical features, etiologies, tailored evaluation, and management for the patient with traumatic Brown syndrome.

## Introduction

Brown syndrome is a rare ocular motility disorder characterized by a limited elevation in the adduction of the affected eye [1]. Brown syndrome may be present at birth (congenital) or may occur as the result of another underlying disorder (acquired) [2]. Congenital causes include shortening or thickening of the tendon sheath of the superior oblique muscle, anomaly in the trochlea, and rare cases of autosomal dominant inheritance [2]. Moreover, acquired Brown syndrome can be caused by trauma, surgery, neoplasm, systemic inflammatory disease, infection, and iatrogenic processes [2]. The forced duction test for patients with Brown syndrome reveals substantial mechanical limitation when attempting to elevate the adducted eye but no such restrictions in abduction [2]. The treatment of Brown syndrome depends on the cause. Some patients may improve spontaneously without intervention, but others require surgical or medical management, such as steroid or nonsteroidal anti-inflammatory treatments [3].

## Case presentation

**Figure 1:**
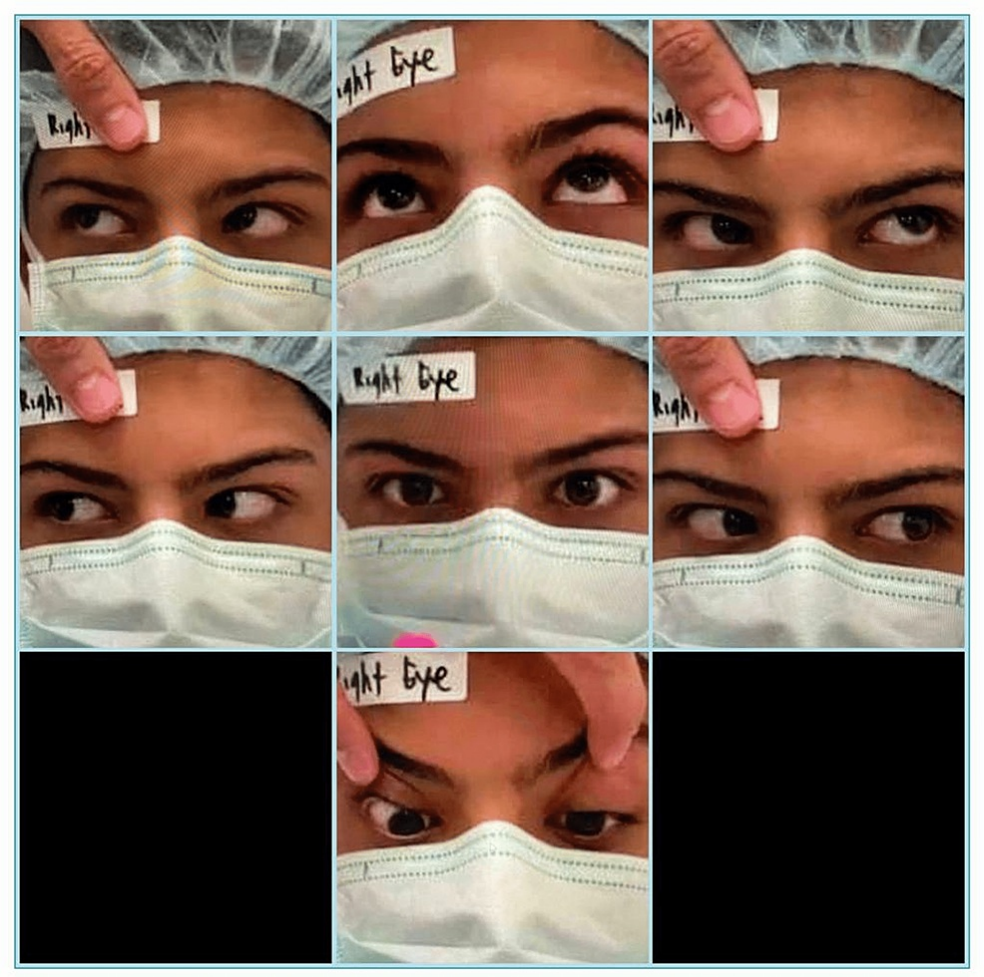
Preoperative orthoptic assessment

**Figure 2:**
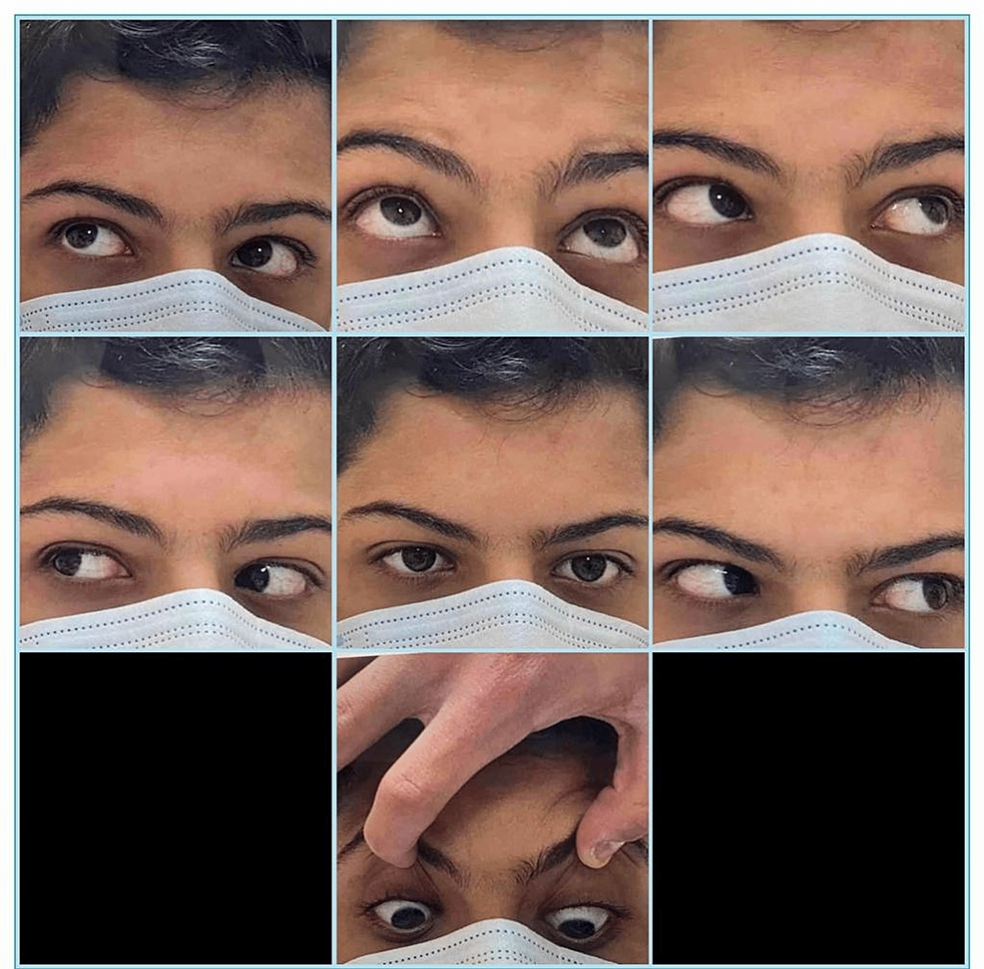
Postoperative orthoptic assessment

A 15-year-old male was hit in the right orbital region by a fist punch during karate practice with his trainer two years prior. He presented to the Emergency Department with a lid ecchymosis, and normal eye examination - a computerized tomography scan of the orbit was performed and showed no orbital wall fractures. One year later, he presented to the ophthalmology clinic with double vision in the left and upward gaze. On examination, he was found to have good vision in both eyes with uncorrected visual acuity of 20/20. He adopted a mild face turn to the left side with a chin-up position. He had limitation of right eye elevation in adduction position around -2 with full motility of the left eye. Slit lamp examination was completely normal in both eyes, and cycloplegic refraction was not significant. The fundus exam was normal in both eyes. The preoperative orthoptic evaluation showed orthotropia in the primary position and right hypotropia of 20 prism diopter (PD) in the left gaze; right hypotropia of 10 PD in the upward gaze; and orthotropia in the right and down gaze (Figure 1). He was diagnosed with the right traumatic Brown syndrome, and a superior oblique lengthening procedure of the right eye was planned. Intraoperatively, the force duction test showed limitation of right eye elevation in the adduction position compared to the left eye. Surgery was performed through a superonasal conjunctival fornix incision between the superior and medial rectus muscles. The superior oblique tendon was hooked with a small muscle hook, and dissection of the intramuscular attachments was carried out. The superior oblique muscle was divided into proximal and distal parts in a Z shape to extend and lengthen the superior oblique tendon. An orthoptic assessment carried out three months postoperatively showed right hypotropia of 5 PD in the left gaze and orthotropia in the upward gaze. The extra-ocular motor function had returned to normal, and the patient was symptomatically much better with the elimination of diplopia and significant improvement of elevation of the right eye in the adduction position (Figure 2).

## Discussion

Brown syndrome is one of the special forms of strabismus. It was first believed to be due to deficit innervation of the inferior oblique with contracture of the superior oblique tendon [4]. It was redefined to include a variety of causes that affect the superior oblique tendon trochlea complex [5]. The congenital form of brown was first described in the early 1970s, with many reports in the literature indicating resistance to spontaneous recovery [5]. Many diseases can contribute to the acquired Brown syndrome, for example, peritrochlear scarring and adhesion, tendon trochlear complex inflammation, direct or indirect trauma, and tight inelastic superior oblique muscle. Unlike the congenital form of Brown syndrome, the acquired form showed better spontaneous recovery [6,7]. Brown syndrome presents as deficient elevation on adduction, with hypotropia in severe cases leaving the affected patient to compensate chin up ahead position with an ipsilateral head tilt [8]. With an attempt on elevation, patients with Brown syndrome can develop V pattern divergence; this considers a good differentiation between Brown syndrome and inferior oblique palsy, which shows A pattern on elevation [8]. Another important exam in diagnosing Brown syndrome is the positive force duction test indicating a restrictive nature of the disease [8].

Some cases of the acquired Brown syndrome after blunt trauma with no proven fracture have been reported. Baker et al. reported two cases that developed Brown syndrome post blunt trauma, one reported full resolution after three weeks, and the latter needed surgical intervention [9]. Khalil et al. reported a seven-year-old who developed Brown syndrome after two months of blunt trauma and received a course of oral syrup of ibuprofen [10]. The child was called for follow-up after one month, showed improvement in subsequent visits, and completely recovered after three months [10]. Our case has presented after one year of blunt trauma, which makes no benefit of any medical treatment because the inflammation has subsided after a long duration of trauma. Also, our case did not show any improvement over two years and needed a surgical intervention to resolve the patient's complaint. Wilson et al. reported 13 cases of Brown syndrome post-trauma were observed over 9-30 months before surgical intervention, with many cases undergoing multiple surgical corrections [8].

In managing patients with Brown syndrome, we rely on the underlying etiology [3]. Although many cases could improve spontaneously, oral nonsteroidal anti-inflammatory drugs or local steroid injection could improve peritrochlear inflammation leading to improvement in Brown syndrome symptoms, especially in cases with active inflammation such as post-traumatic and rheumatoid arthritis. Surgical management of Brown syndrome is usually reserved for those who develop chin elevation, severe elevation deficit on adduction, or interfere with their daily life activity [3]. Surgical treatment for Brown syndrome has shifted, with sheathectomy and superior oblique trochlear luxation having been proven their unsatisfactory result [8]. Superior oblique weakening procedure, such as superior oblique tendon lengthening, tendon expander technique, tenotomy, and superior oblique recession, is the surgery of choice in cases with acquired Brown syndrome [8].

## Conclusions

Traumatic Brown syndrome is an unusual presentation after a fist punch. Surgical intervention is indicated if the Brown syndrome is not resolved after 9-12 months post-trauma. Superior oblique weakening procedures are expected to have good outcomes.
